# Enhancing the Combustion of Magnesium Nanoparticles via Low-Temperature Plasma-Induced Hydrogenation

**DOI:** 10.1021/acsami.3c12696

**Published:** 2023-10-30

**Authors:** Brandon Wagner, Minseok Kim, Mahbub Chowdhury, Emmanuel Vidales Pasos, Kimberly Hizon, Pankaj Ghildiyal, Michael R. Zachariah, Lorenzo Mangolini

**Affiliations:** † Materials Science and Engineering Program, University of California Riverside, 900 University Avenue, Riverside, California 92521, United States; ‡ Department of Mechanical Engineering, University of California Riverside, 900 University Avenue, Riverside, California 92521, United States; § Department of Chemical and Environmental Engineering, University of California Riverside, 900 University Avenue, Riverside, California 92521, United States

**Keywords:** magnesium, nonthermal plasma, hydrogen treatment, magnesium hydride, combustion, ignition, energetics

## Abstract

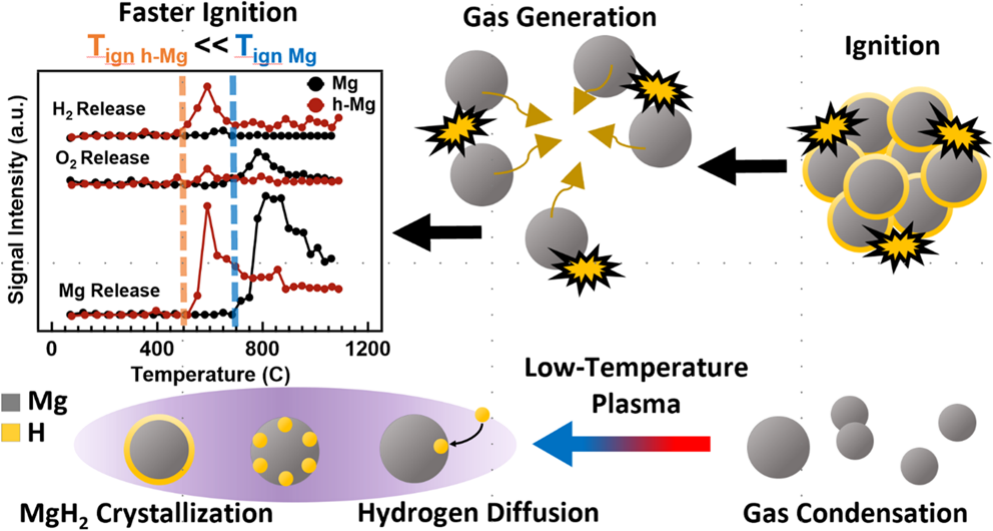

The hydrogenation of metal nanoparticles provides a pathway toward tuning their combustion characteristics. Metal hydrides have been employed as solid-fuel additives for rocket propellants, pyrotechnics, and explosives. Gas generation during combustion is beneficial to prevent aggregation and sintering of particles, enabling a more complete fuel utilization. Here, we discuss a novel approach for the synthesis of magnesium hydride nanoparticles based on a two-step aerosol process. Mg particles are first nucleated and grown via thermal evaporation, followed immediately by in-flight exposure to a hydrogen-rich low-temperature plasma. During the second step, atomic hydrogen generated by the plasma rapidly diffuses into the Mg lattice, forming particles with a significant fraction of MgH_2_. We find that hydrogenated Mg nanoparticles have an ignition temperature that is reduced by ∼200 °C when combusted with potassium perchlorate as an oxidizer, compared to the non-hydrogenated Mg material. This is due to the release of hydrogen from the fuel, jumpstarting its combustion. In addition, characterization of the plasma processes suggests that a careful balance between the dissociation of molecular hydrogen and heating of the nanoparticles must be achieved to avoid hydrogen desorption during production and achieve a significant degree of hydrogenation.

## 1 Introduction

Magnesium hydride (MgH_2_) is a potential solid-state hydrogen storage material because of its high capacity (7.6 wt %), which exceeds the standards set by the U.S. Department of Energy for hydrogen storage materials.^1−4^ MgH_2_ is also a promising candidate for combustion-related applications due to its high combustion enthalpy and hydrogen content. For instance, metal hydrides are attractive additives in solid rocket propellants since they have been shown to improve combustion performance.^5,6^ Oxidation of hydrogen gas (H_2_) is highly exothermic, contributing to the overall energy release during the ignition of solid metal fuels.^7^ Xi et al. found that combining metal hydrides with boron particles enhances the ignition by releasing heat, thus increasing the temperature of boron and facilitating its burning.^8^ The effects of titanium hydride (TiH_2_), zirconium hydride (ZrH_2_), and MgH_2_ on combustion were studied by Fang et al., in which the addition of the metal hydrides resulted in more intense flames and higher combustion rates.^9^ Particularly, the sample with MgH_2_ as an additive had the highest combustion rate compared to those of TiH_2_ and ZrH_2_. Young et al. investigated the ignition behavior of aluminum hydride (AlH_3_) microparticles and found that their ignition threshold was considerably lower than aluminum (Al) microparticles.^10^ Micrometer-sized AlH_3_ exhibited ignition behavior similar to nanoscale Al particles. Among the existing metal hydrides, MgH_2_ is particularly attractive for fuel additives because of its high hydrogen storage capacity, along with it releasing H_2_ gas and magnesium (Mg) vapor during ignition, generating large amounts of pressure.^11,12^

Particle agglomeration and sintering are common issues when using nanosized particles for ignition purposes.^13^ Previous studies have suggested that gas generation during the ignition of nanoparticles can alleviate these problems by propelling particles apart, preventing agglomeration, and allowing the complete combustion of the nanoparticle fuel.^14−16^ Young et al. explored aluminum-based mesoparticles loaded with a gas-generating binder, nitrocellulose, and found that sintering was minimized during ignition to allow full combustion of the particles.^17^ For biocidal purposes, gas generators are important for dispersing a biocidal to the deactivation area of biological weapons.^18^ To summarize, the hydrogenation of nanofuels is beneficial for both combustion kinetics and gas generation, thus providing an additional handle for the optimization of energetic formulations.

Several methods have been implemented to prepare MgH_2_. Mechanochemical ball milling is a typical approach to prepare MgH_2_ from a Mg precursor; however, this method generally requires high pressures (several MPa) and relatively high temperatures exceeding 300 °C while using catalysts.^19−21^ Additionally, lengthy production times are necessary for complete hydrogenation by this process. Plasma-assisted ball milling is an enhanced technique that allows the production of Mg-based alloys containing MgH_2_ for hydrogen storage applications. The use of plasma enables electron and ion bombardment of the metal precursors that facilitates the formation of hydrogen storage alloys in shorter processing times.^22−24^ The thermodynamics and kinetics of H_2_ desorption can be tuned by alloying other metals with Mg.^25^ Ouyang et al. synthesized a MgF_2_ doped Mg–In alloy to maintain a high hydrogen storage capacity while lowering the activation energy for H_2_ desorption.^26^ Dan et al. produced MgH_2_–Ni composites through a similar method and found that dehydrogenation can occur at temperatures as low as 225 °C.^27^ MgH_2_ films have been produced by plasma-assisted physical vapor deposition, in which synthesis occurs in a low-pressure environment.^28,29^ The process involves lower temperatures and shorter hydrogenating times compared with other methods.

Nonthermal plasmas can process various materials at near room temperature and low pressures (1–10 Torr).^30−35^ Free electrons with temperatures in the 1–5 eV range can activate a broad range of chemistries, even while the gas remains close to room temperature, including in the case of the nucleation of nanoparticles. An additional advantage of low-temperature plasmas (LTPs) is the electrostatic stabilization of nanoparticles dispersed within them.^36,37^ This effect prevents agglomeration, allowing the functionalization of individual particles on-the-fly, as opposed to agglomerates. For example, LTPs have been used to apply surface coatings on nanoparticles in-flight to produce a conformal Si-based shell around Mg core particles for accelerated ignition.^38^ Silicon NPs synthesized by LTPs are widely studied materials. Xu et al. produced crystalline and amorphous Si NPs by varying the plasma power and studied their effects on ignition.^39^ Because amorphous Si contains more hydrogen-terminated Si on the surface, more pressure generation was observed due to increased amounts of H_2_ gas released from the particles. Other work shows that Al NPs can be treated with hydrogen to dope the oxide layer with aluminum hydride (AlH_3_).^40^ The results showed that H_2_ gas generation increased the heat release during combustion by exposing more aluminum to react with the oxidizer upon heating.

In this work, Mg NPs produced by the gas-condensation method are subjected to in-flight hydrogen plasma treatment to synthesize MgH_2_-containing (h-Mg) NPs. To the best of our knowledge, there are no reports of MgH_2_ produced by LTPs for combustion applications. The effects of plasma processing on the hydrogen content and ignition kinetics are investigated. Process characterization suggests that in-flight hydrogenation is due to the high density of atomic hydrogen achieved by low-temperature plasma. Diffusion of atomic hydrogen into the magnesium lattice results in the formation of particles with MgH_2_ in their outer layer, as confirmed by careful transmission electron microscopy analysis. Combustion was tested against Mg NPs produced without plasma treatment with a potassium perchlorate (KClO_4_) oxidizer to examine the effects of H_2_ gas release on ignition. The results discussed in this contribution show that H_2_ gas release reduces the ignition threshold of the nanothermite mixtures, consequently enabling the faster release of the Mg fuel for combustion.

The manuscript is organized as follows: after Section 2, we provide extensive characterization of the nanomaterials by X-ray powder diffraction (XRD), scanning electron microscopy (SEM), and high-resolution transmission electron microscopy (TEM) in Section 3.1. The influence of MgH_2_ formation on its combustion performance is detailed in Section 3.2. Additional characterizations of the plasma process are given in Section 3.3.

## 2 Methods

### 2.1 In-Flight Hydrogen Treatment of Magnesium Nanoparticles

**Figure 1:**
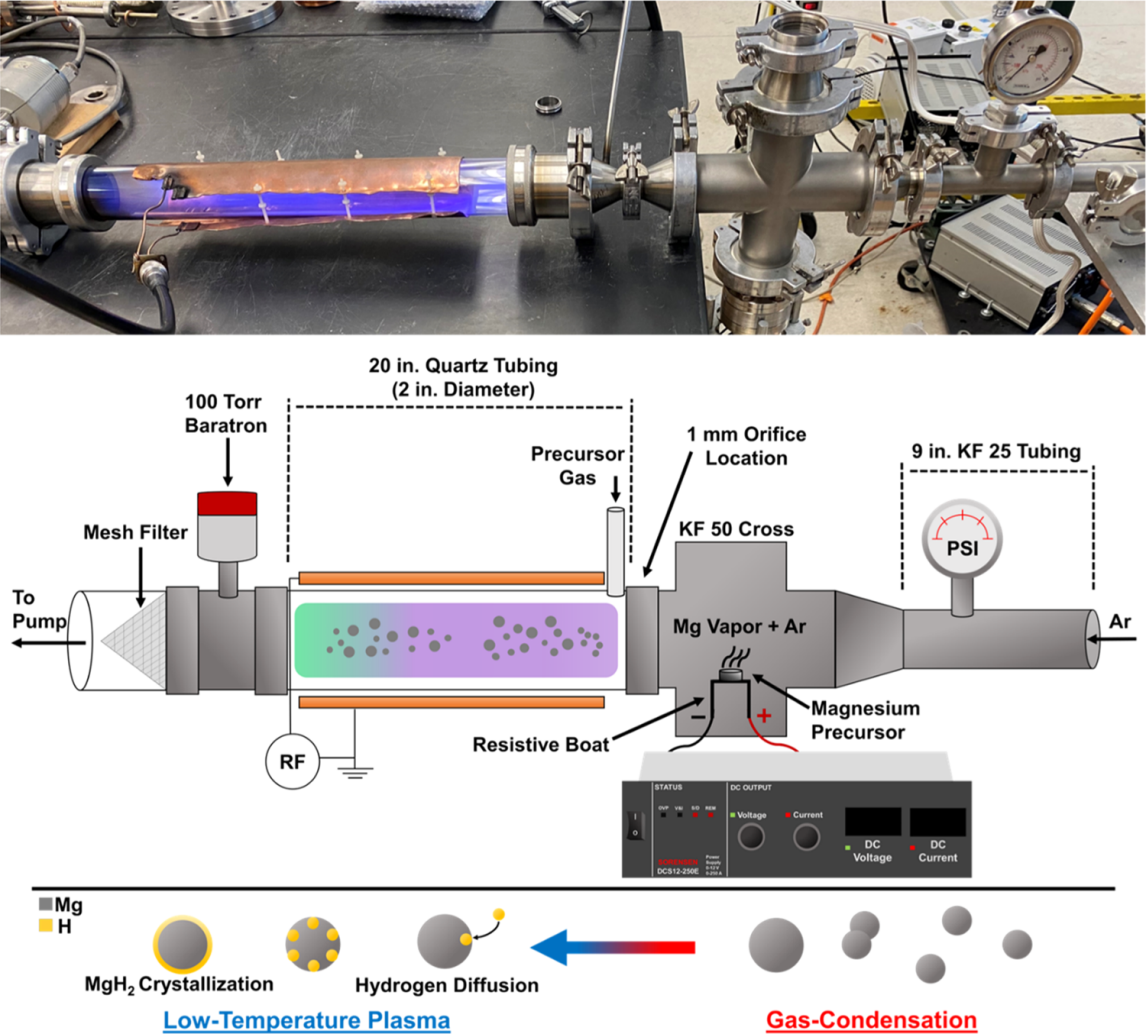
Photograph and schematic of the thermal evaporation and nonthermal hydrogen plasma system.

Magnesium NPs were prepared by the gas condensation of Mg vapor and exposed to a low-temperature hydrogen plasma for hydrogenation, as shown in Figure 1. Bulk Mg precursor (∼250 mg, disc shape, 1.2 cm diameter, and 1 mm thickness) was resistively heated while high-purity argon (Ar) was flown (350 sccm) as a carrier gas. The pressure in the evaporation chamber was ∼40 Torr. The Mg-laden aerosol was injected into the plasma reactor by flowing through an orifice while high-purity H_2_ gas was introduced at 30 sccm for hydrogen treatment. The plasma reactor consisted of 2” × 20” quartz tubing and copper parallel plate electrodes connected to a radio frequency (RF) power supply and matching network. The RF power at 13.56 MHz was varied, while the pressure was maintained at 3 Torr in the plasma reactor. Based on flow velocity, the estimated residence time of the Mg-laden aerosol in the plasma reactor was ∼185 ms. The samples were collected downstream of the plasma reactor onto a stainless-steel mesh filter in vacuum. The NPs were removed from the vacuum system by slowly leaking air to prevent ignition.

### 2.2 Preparation of Nanothermite Composites

Mg and h-Mg powders were mixed with the KClO_4_ oxidizer in hexanes and ultrasonicated to obtain homogeneous mixtures. The mixtures were dried under ambient conditions for 24 h for collection. Each fuel sample was prepared with stoichiometric equivalents of the KClO_4_ oxidizer (fuel:oxidizer equivalence ratio, ϕ = 1) and mixed into nanothermite composites.

### 2.3 T-Jump/TOFMS and Ignition Characterization

Temperature-jump time-of-flight mass spectrometry (T-jump/TOFMS) was used to investigate the gaseous products produced during ignition, as well as their time-resolved release and ignition temperature. The sample mixtures were coated onto a platinum (Pt) wire and resistively heated with a rapid pulse (∼3 ms) to ∼1200 °C, which resulted in a high heating rate of ∼10^5^ °C s^–1^. The current applied to the Pt wire was measured using a Teledyne LeCroy CP030A current probe to obtain the wire temperature, which is attained from the current–voltage relationship from the Callendar–Van Dusen equation. Gaseous products created from ignition reactions were ionized using a 70 eV electron gun and were accelerated toward a multichannel plate detector maintained at ∼1500 V. Mass spectra were recorded with a high temporal resolution (0.1 ms) to probe the relevant time scales of fast combustion reactions (∼1 ms). The T-jump/TOFMS instrument was equipped with a high-speed camera (Vision Research Phantom V12.1) to record the ignition events.

### 2.4 Material Characterization

X-ray diffraction (XRD) was performed (PANalytical Empyrean Series 2) to analyze the crystallinity and composition of the samples. Curve fitting by the Rietveld refinement method was used using Profex (version 5.2.0) software in order to obtain semiquantitative material composition of MgH_2_ and Mg composition within the h-Mg samples. Particle morphology was analyzed using a ThermoFisher Scientific NNS450 scanning electron microscope (SEM) using a 15 kV accelerating voltage. An FEI Titan Themis 300 transmission electron microscope (TEM) was used to obtain high-resolution transmission electron microscopy (HR-TEM) images. The TEM grids were prepared by ultrasonicating the powder samples in isopropanol for 2 min and drop-casting the dispersed solution onto lacey-carbon grids. The average size and size distributions were obtained by measuring 1000 particles from each sample from TEM images in ImageJ software (version 1.53k). Temperature-programmed desorption (TPD) was performed to verify hydrogen content in the nanopowders. About 50 mg of h-Mg powder was loaded onto an alumina boat and placed into the center of a 1-inch quartz tube and heated using a furnace *f* to 550 °C with a 10 °C min^–1^ heating rate. A residual gas analyzer (RGA) was used to determine the composition of the gas downstream of the heated region. The hydrogen signal at amu = 2 was carefully corrected by removing the contribution from moisture. This was done by acquiring a background mass spectrum with argon flowing through the system to determine the ratio between the water signal at amu = 18 and the hydrogen signal at amu = 2 from the water dissociation. Thermogravimetric analysis (TGA) and differential scanning calorimetry (DSC) were performed by using a Netsch STA449 F3 Jupiter analyzer to monitor the slow oxidation of NPs with a heating rate of 10 °C min^–1^.

### 2.5 Plasma Characterization

Optical emission spectroscopy (OES) was used to characterize the excited species in the plasma based on the light emitted during processing. A fiber-optic cable was positioned perpendicular to the plasma reactor tube to collect light and pass it to a monochromator (Princeton Instruments, Acton SpectraPro SP-2750). Emission spectra are acquired and processed by LightField software. Plasma emission from 500 to 800 nm is measured to obtain the emission line intensities with respect to Mg, H_2_, and Ar.

## 3 Results and Discussion

### 3.1 Characterization of Synthesized Nanoparticles

**Figure 2:**
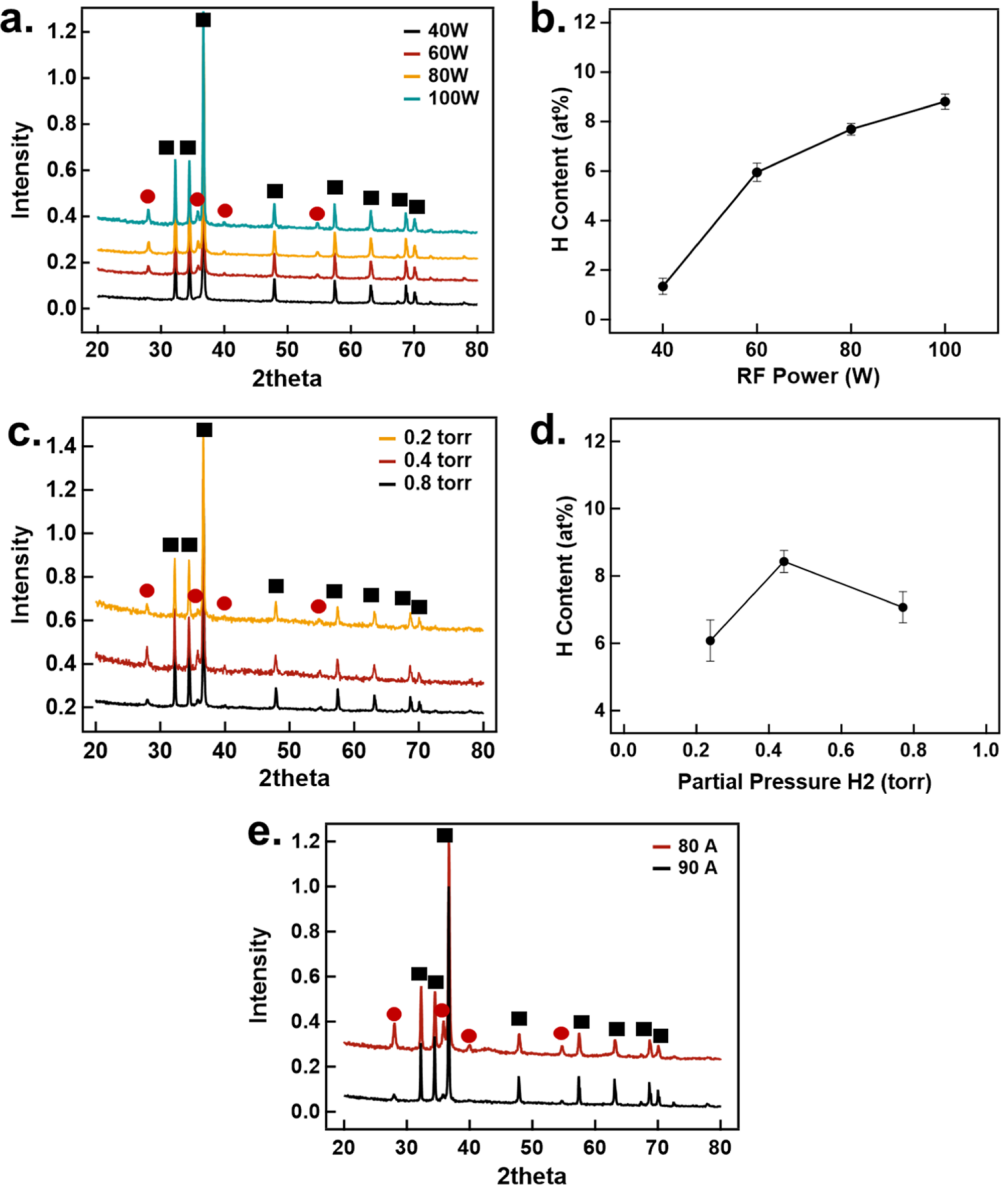
XRD patterns of h-Mg NPs produced using (a) an RF power of 40–100 W and (c) partial pressure of H_2_ and their respective H content (b, d). XRD patterns of nanoparticles made with low (80 A) and high (90 A) currents are shown in panel (e). Red circles denote MgH_2_ planes, and black squares denote Mg planes in the XRD patterns.

XRD was used to analyze the composition and crystallinity of the h-Mg NPs synthesized by various RF powers (Figure 2a) and partial pressure of H_2_ (Figure 2c). Rietveld refinement was used to determine the Mg and MgH_2_ composition according to the RF power and partial pressure of H_2_ in weight percent, from which the atomic percentages of H were calculated (Figure 2b,2d). Increasing RF power from 40 to 100 W raises the overall H content within the h-Mg NPs from 2 to 8 atom %, with the increase leveling off at a high RF power. This confirms the ability of the low-temperature plasma to achieve substantial hydrogenation of the Mg particles. The leveling off at a higher RF power and plasma intensity will be discussed in detail later in the manuscript. It is likely the consequence of nanoparticle heating inducing hydrogen desorption and limiting the hydrogen content in the material. With respect to gas composition, an optimal H_2_ partial pressure of 0.4 Torr produces the largest degree of hydrogenation at a constant power of 100 W. Increasing the flow of molecular hydrogen beyond this level is actually detrimental. This result is not surprising, as an excessive fraction of H_2_ gas in the plasma is expected to quench it due to energy transfer to vibrational and rotational excitation of H_2_. TPD was used to assess the accuracy of the MgH_2_ content obtained through the Rietveld refinement fitting method. A sample consisting of 16.7 atom % MgH_2_ was produced using 82 A of current and an RF power of 80 W, containing 0.372 mmol of H_2_ for 55 mg of powder. TPD showed that the volume of H_2_ desorbed from the particles was 7.9 cm^3^ (Figure S1). The volume was calculated by integrating the area underneath the flow rate vs. time plot, which equated to 0.355 mmol of H_2_ and is in reasonable agreement with the amount obtained by Rietveld refinement. More details can be found in the Supporting Information regarding the estimation of H_2_ mmol from the sample. The results show that the Rietveld refinement is a reliable technique to estimate the fraction of MgH_2_ within the h-Mg NPs, from which the H content can be calculated. We have also found that the overall production rates of the h-Mg NPs are strongly dependent on the RF power (Figure S2a). When the power is 40, 60, 80, and 100 W, the production rates are 16, 8, 7, and 6 mg h^–1^, respectively. The production rate drops considerably at higher plasma power. Figure S2b shows the plasma reactor tube before synthesis. After synthesis, there is noticeable film growth onto the plasma reactor tube at 80 and 100 W of RF power (Figure S2c,d). The high amount of film growth at a high RF power is indicative of Mg losses to the walls of the reactor, consistent with the production rate of h-Mg NPs being strongly dependent on plasma power. Finally, we have found that the MgH_2_ content in the NPs is strongly dependent on the particle size. Reducing the current in the evaporation step from 90 to 80 A results in nanoparticle sizes decreasing from 400 to 130 nm on average, respectively (Figure S3a,b). 500 particles were measured for both samples in ImageJ software from SEM images to obtain the average sizes. The corresponding XRD patterns are shown in Figure 2e. Rietveld refinement indicates that the atomic fraction of MgH_2_ increases from 7.4 to 23.5 atom % when decreasing particle size.

**Figure 3:**
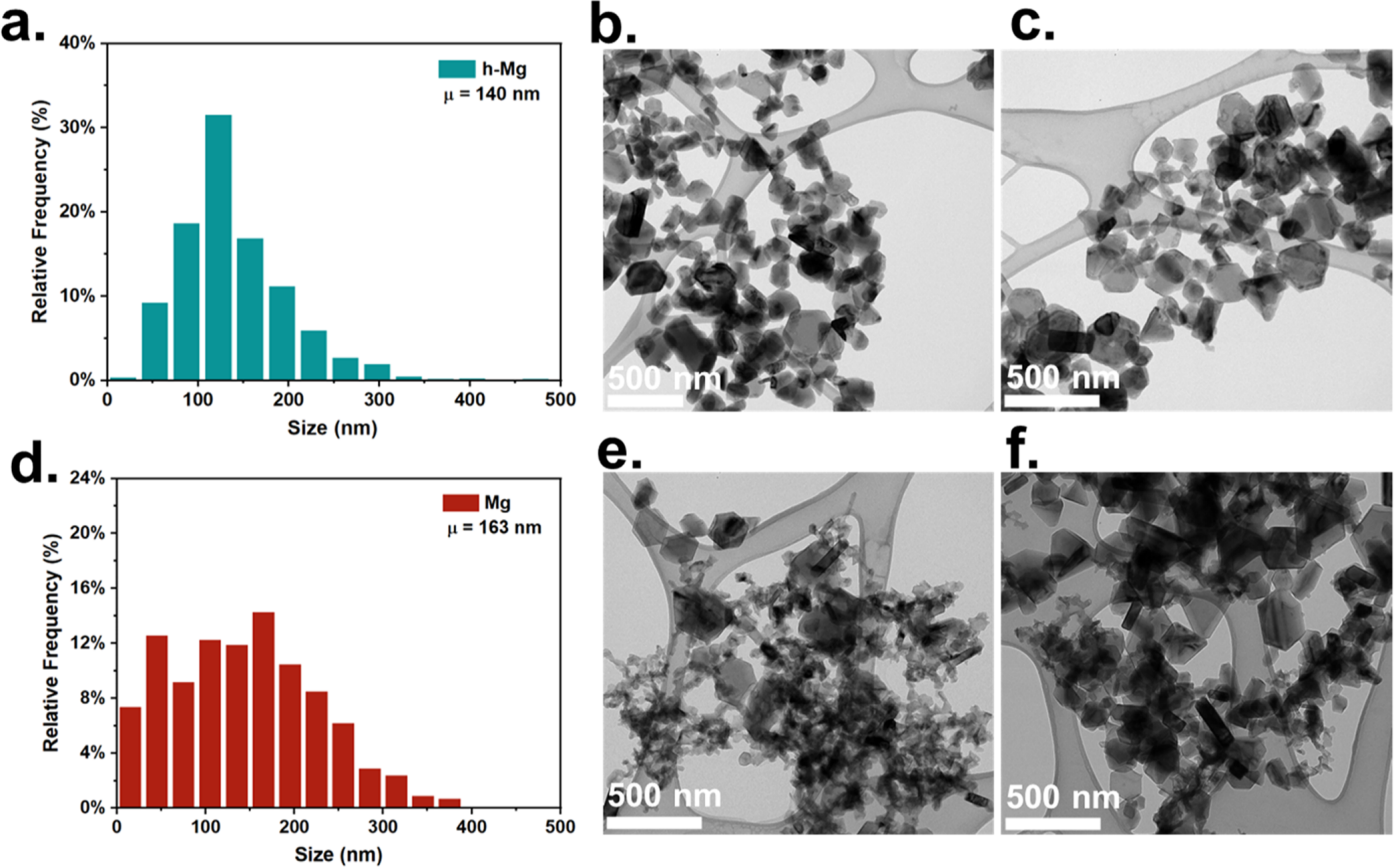
(a) Size distribution of 1000 h of Mg NPs from TEM. (b, c) TEM images representative of the size distribution of h-Mg NPs. (d) Size distribution of 1000 Mg NPs from TEM. (e, f) TEM images representative of the size distribution of Mg NPs.

The morphology and size of the Mg and h-Mg NPs were analyzed by TEM. The size distributions of h-Mg and Mg NPs with their respective TEM images are shown in Figures 3a–c and 3d–3f, respectively. Both samples have hexagonal crystal morphology, which is the thermodynamically most favorable structure for Mg.^41^ Mg and h-Mg have similar average sizes (∼150 nm), but the size distributions are dissimilar. Interestingly, the Mg NPs have wider size distributions than h-Mg NPs, signifying that exposure to hydrogen plasma results in focusing the NPs to narrower size distributions. Similar observations were found for plasma-treating bismuth particles in-flight, in which the particle size distribution narrowed after plasma exposure.^42^ There are noticeably fewer NPs below 50 nm upon hydrogen treatment, possibly due to their evaporation within the plasma. Mg NPs of 40 nm have shown an evaporation temperature of about 800 K,^43^ which particle temperatures in the plasma can reach 800 K when operating at 80 W of RF power, as discussed later in the manuscript.

**Figure 4:**
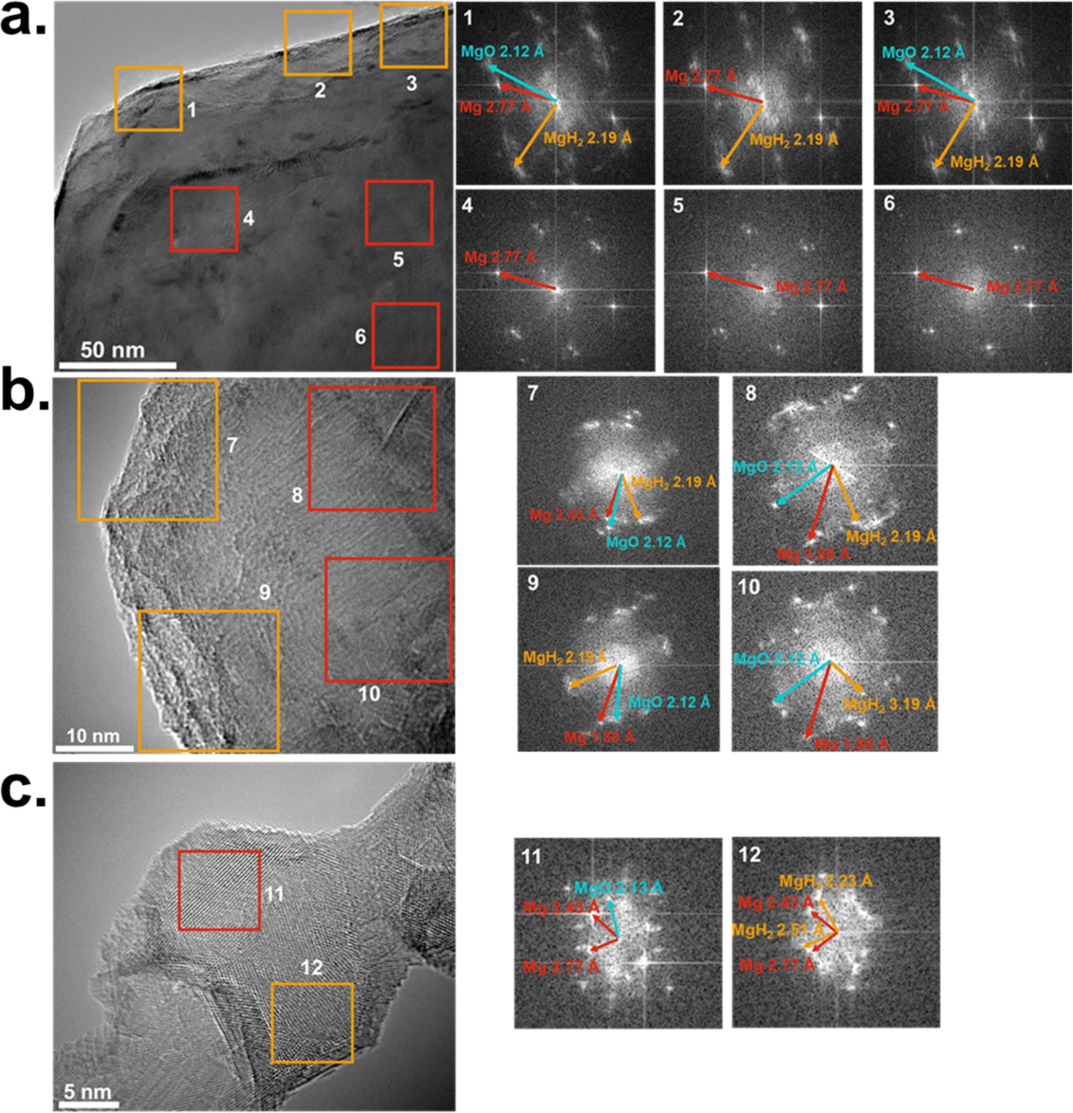
HR-TEM of (a) a 300 nm particle, (b) a 100 nm particle, and (c) a 30 nm particle, along with their respective FFT diffraction patterns. Blue arrows indicate the MgO lattice plane; yellow arrows indicate the MgH_2_ lattice plane; and red arrows indicate the Mg lattice plane.

HR-TEM was used to gain insight into MgH_2_ growth on the Mg NPs when exposed to a hydrogen plasma. Particles of various sizes were analyzed, showing the presence of MgH_2_ at different areas on the Mg crystals according to size. The TEM images of h-Mg crystals with particle sizes of 50–300 nm are shown along with their respective FFT diffraction patterns in Figure 4a–c. For ∼300 nm particles, the surface consists of an ∼45 nm thick MgH_2_ layer, whereas the bulk shows only Mg. For particle sizes of ∼100 nm, both the surface and bulk of the Mg crystals contain MgH_2_. Particles of ∼50 nm also have MgH_2_ within the bulk, but parts of the crystal lack hydrogenation. Nonthermal plasmas are notorious for generating reactive gas species due to electron collisions, whereby the precursor radicals would react with the surface of nanoparticles or thin films. In the case of hydrogenation, atomic hydrogen generated by the LTP using a H_2_ gas precursor can react with the Mg NPs aerodynamically carried through the plasma reactor. The atomic H diffuses into the Mg particle to react and nucleate MgH_2_, forming crystalline domains and creating a hydride outer layer. Remarkably, MgH_2_ is prevalent throughout the entire crystal for 100 nm particles, suggesting that smaller NPs are easier to hydrogenate than larger ones. Previous work has found that the uptake of hydrogen into bulk Mg is a diffusion-controlled process through MgH_2_ at the surface. The diffusion coefficient (*D*) of hydrogen into Mg is 10^–13^ m^2^ s^–1^ at 300 K compared to 10^–17^–10^–20^ m^2^ s^–1^ through MgH_2_.^11,44^ Therefore, hydrogen diffusion is considerably slower through MgH_2_ than Mg. Using the residence time of the particles in the plasma (∼185 ms) and Fick’s diffusion law, we estimate the hydrogen diffusion length in Mg to be ∼105 nm. This value is consistent with the thickness of the MgH_2_ layer in the larger particles (∼45 nm). The apparent difference between the estimated diffusion length and the actual thickness of the MgH_2_ layer is likely due to the decrease in diffusivity once the MgH_2_ lattice is formed. The fact that the MgH_2_ formation is diffusion-limited is also consistent with the results shown in Figure 2e, with smaller nanoparticles having a higher fraction of MgH_2_ compared to larger ones. As a whole, the combination of XRD, SEM, and HR-TEM results suggests that particle size must be minimized to achieve a high degree of hydrogenation. On the other hand, other effects, such as nanoparticle heating and hydrogen desorption, play a role in the plasma-induced hydrogenation process. These effects are described in greater detail in Section 3.3.

### 3.2 Ignition Characterization

**Figure 5:**
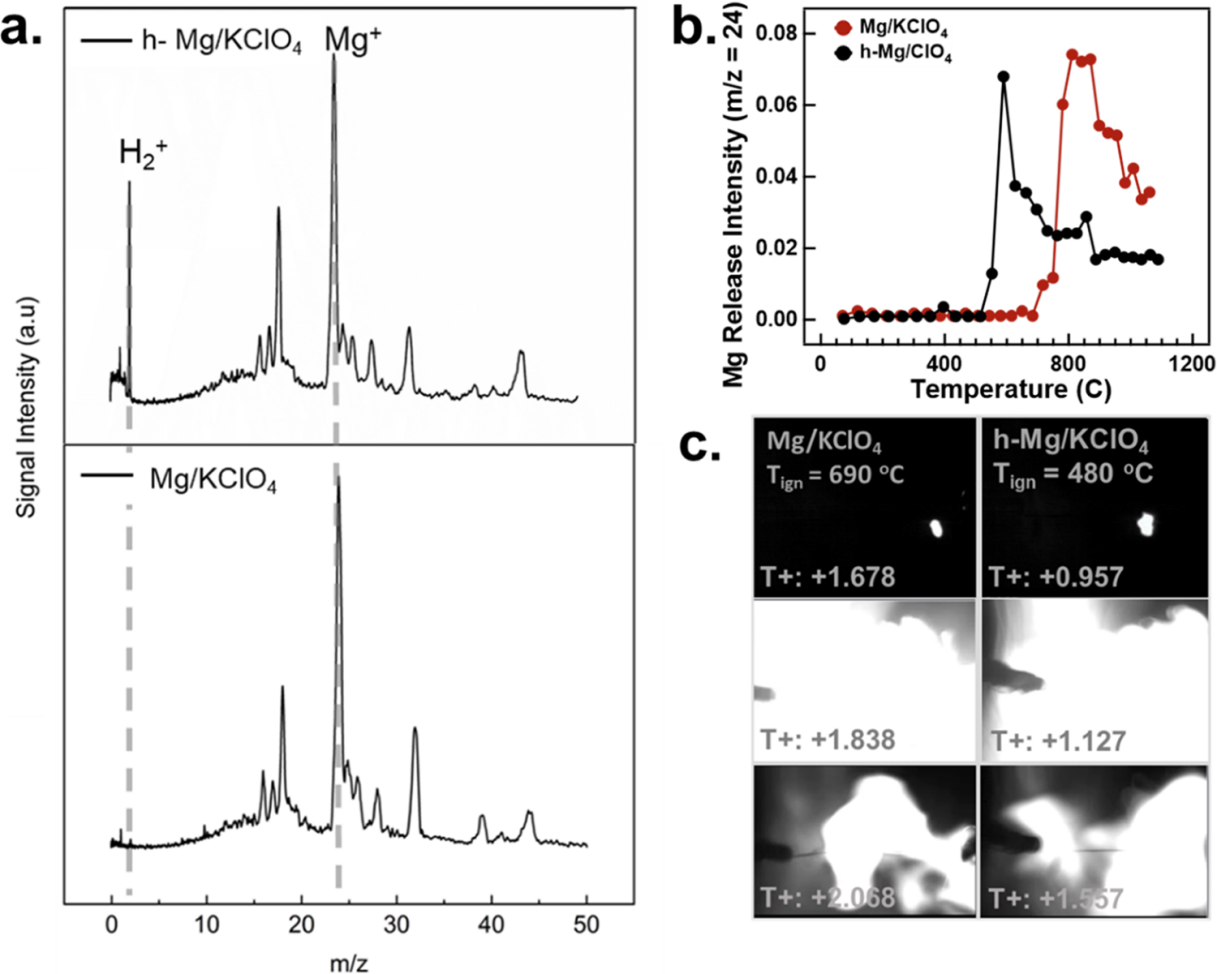
(a) Mass spectrometry spectra of h-Mg and Mg NPs and (b) Mg release profiles from T-jump/TOFMS for h-Mg/KClO_4_ and Mg/KClO_4_. (c) High-speed camera images of ignited Mg/KClO_4_ and h-Mg/KClO_4_ thermite mixtures.

The reaction species of the samples during ignition were characterized using T-jump/TOFMS in an Ar environment. KClO_4_ was used as the oxidizer because it releases oxygen at a low temperature (KClO_4_ ∼ 580 °C).^43^ The ignition mechanism can be investigated by comparing the ignition temperature and time from the sample/oxidizer mixtures to the O_2_ release temperatures of the oxidizer. If O_2_ releases after ignition, then the relevant reaction mechanism occurs in the solid state instead of a gas-phase reaction when O_2_ releases beforehand. The T-jump/TOFMS results of h-Mg and Mg are shown in Figure 5a, displaying similar spectra, except H_2_ is detected for h-Mg NPs. Samples were combusted with the KClO_4_ oxidizer in the T-jump/TOFMS instrument to determine the Mg release profile according to the temperature and time (Figures 5b). Mg releases earlier from h-Mg NPs when reacting with KClO_4_ compared with Mg NPs. T-jump measurements showed that the ignition temperatures for h-Mg/KClO_4_ and Mg/KClO_4_ mixtures were ∼480 and ∼690 °C, respectively. A high-speed camera was used to record optical emission during ignition to help obtain ignition temperatures of the thermite mixtures, as shown in the time-stamped images in Figure 5c. These results indicate that the incorporation of H_2_ in the Mg NPs results in lower ignition thresholds with a drastically reduced ignition temperature by over 200 °C when reacting with KClO_4_. H_2_ in h-Mg NPs facilitates the release of Mg at a lower temperature, making the fuel accessible at a lower temperature and leading to faster ignition.

**Figure 6:**
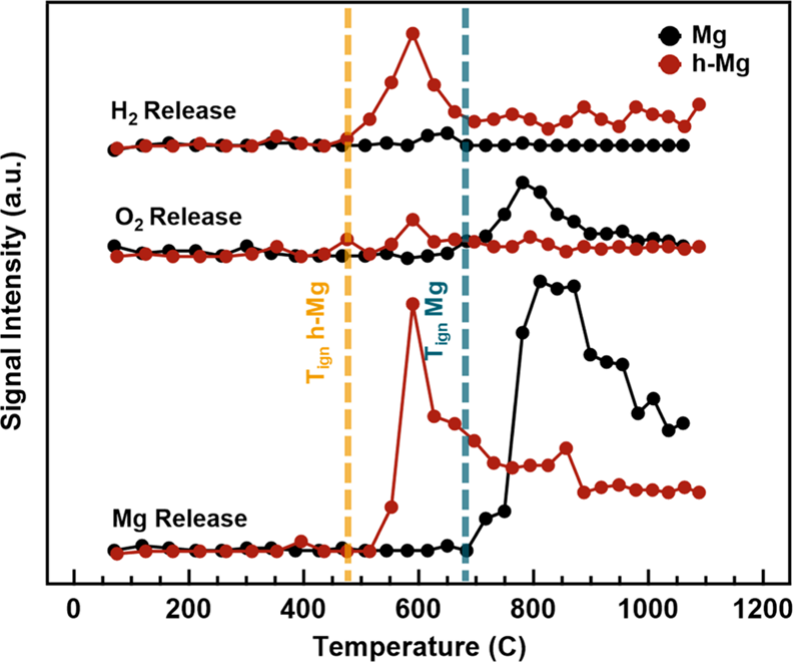
Hydrogen, oxygen, and magnesium release profiles from T-jump/TOFMS for h-Mg and Mg NPs during ignition with KClO_4_. The yellow dashed line indicates the ignition temperature of h-Mg NPs, while the blue dashed line denotes the ignition temperature of Mg NPs.

Figure 6 shows the H_2_, O_2_, and Mg release intensities from the T-jump/TOFMS measurements during the ignition of the nanothermite mixtures. The O_2_ release temperature significantly reduces from ∼680 to ∼500 °C for h-MgFor Mg NPs, and ignition occurs slightly after O_2_ is released. This result indicates that the mechanism for Mg with KClO_4_ is controlled by the decomposition of the oxidizer to release gas-phase O_2_. In contrast, the ignition mechanism changes with the addition of MgH_2_. Ignition occurs after the desorption of H_2_ but before the release of O_2_ from KClO_4_. This implies that the presence of H_2_ and its desorption from MgH_2_ controls ignition. Interestingly, the decomposition of KClO_4_ occurs after ignition begins, indicating that a condensed-phase reaction mechanism may take place between MgH_2_ and KClO_4_. Early H_2_ gas generation may have a significant effect on ignition with KClO_4_ because H_2_ can react with O_2_ from the oxidizer, effectively releasing supplemental heat to initiate the release of Mg vapor earlier for accelerated combustion. Equations 1 and 2 show the enthalpies of the MgH_2_ and Mg reactions with KClO_4_.
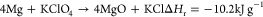
1
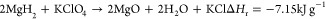
2The gravimetric reaction enthalpy of MgH_2_ with KClO_4_ is lower than Mg; however, T-jump/TOFMS indicates that the reaction kinetics are faster since H_2_ reacts with the oxidizer and initiates Mg release. Another possibility for the greater reactivity of MgH_2_ compared to Mg may be related to changes in the surface chemistry. H_2_ desorption from the particle outer layer likely exposes reactive Mg sites, providing a pathway for oxygen to interact with and accelerate combustion. In general, we can conclude that MgH_2_, which does not increase the energy density based on thermodynamic arguments, offers a pathway to reduce the ignition threshold and acceleration of the overall kinetics.

### 3.3 Plasma Characterization

**Figure 7:**
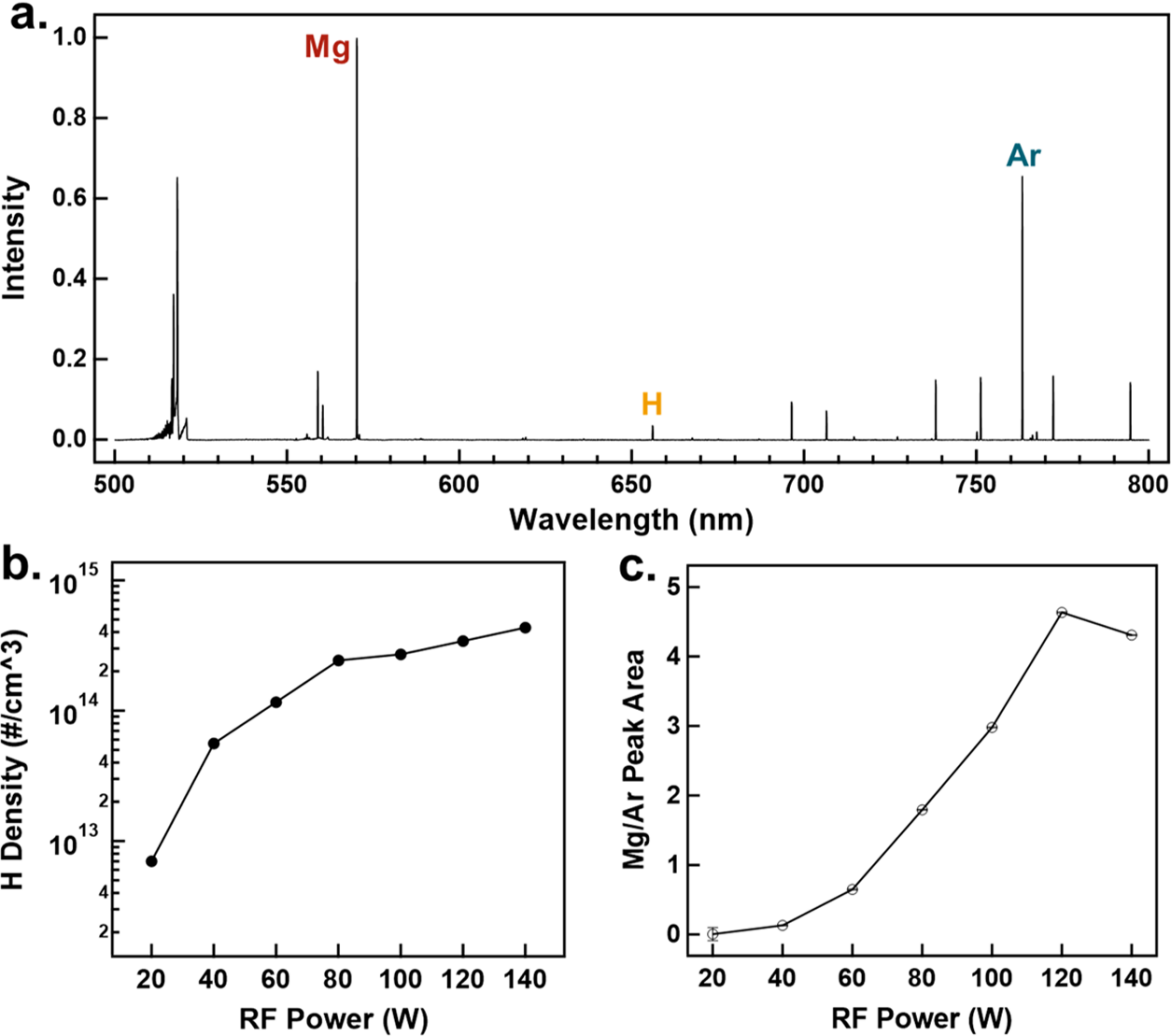
(a) OES spectra of the plasma. (b) Actinometry was used to obtain the atomic H density according to the RF power. The integrated area ratio of the Mg 570 nm and Ar 763 nm lines were obtained from the OES spectra to gain insight into the atomic Mg density at different plasma powers (c).

OES was used to characterize the plasma chemistry. Figure 7a shows the measured OES spectra at 80 W of RF power. As expected, the emission spectrum is rich with many lines. Emission lines from atomic metals H and Ar are clearly observable. The emission line assignment is based on the broadly utilized NIST atomic spectra database.^45^ The Mg emission at 570.4 nm is the most prominent signal from the plasma, followed by the Ar line at 763.51 nm and the H_α_ line at 656.279 nm. We should note that the Mg emission at 570.4 nm is the second-order emission from the 285.2 nm line corresponding to the 3s3p–3s^2^ transition of neutral Mg. As shown in Figures S4a–c, the Mg, Ar, and H emission lines become more intense with increasing RF power, as expected, since the plasma density increases with power. Actinometry is used to estimate the density of atomic hydrogen within the plasma. To that end, we have used the intensity ratio between the H 656 nm and Ar 763 nm lines, coupled with a reasonable estimate of the electron-induced excitation rates as obtained by a commonly utilized Boltzmann solver, Bolsig+.^46^ Details about the approach are given in the Supporting Information. The electron temperature is not known a priori. We assume a reasonable value of 5 eV. The atomic hydrogen density values obtained using this approach are shown in Figure 7b. As expected, the level steadily increases with the RF power. The atomic hydrogen density is considerable, exceeding 10^14^ cm^–3^, as it is typically observed in these midpressure processes. This supports our hypothesis that atomic hydrogen plays a crucial role in the fast hydrogenation of Mg nanoparticles as they travel through the plasma. The conversion of molecular H_2_ to atomic H is a well-known process within LTPs. There are several pathways through which atomic H is generated. Electron impact with rovibrationally excited H_2_ molecules can exceed the energy threshold to break apart the H_2_ bonds. The same process can occur for electronically excited H_2_ bonds.^47−49^ Atomic hydrogen can then readily diffuse into the Mg lattice through the interstitial sites of the hexagonal lattice.^50^

We also note that the Mg atomic line at 570 nm is very intense. While it is possible to estimate the density of atomic magnesium using actinometry, similarly for hydrogen, there are no reported cross-sections for the electron-impact-induced excitation of Mg atoms. Nevertheless, Figure 7c shows the ratio between the Mg 570 nm and the Ar 763 nm peak areas with varying RF power, which is strongly dependent on power. As mentioned earlier in the manuscript, the yield of Mg nanoparticles decreases significantly with power (Figure S2a), and at higher power, we observe the rapid growth of a metallic film onto the reactor’s inner walls. These observations, therefore, suggest that Mg nanoparticles can evaporate as they are exposed to the low-temperature plasma and do so more rapidly at higher RF input power. Ion bombardment could potentially sputter Mg from condensed particles in the plasma; however, the sputtering yield is high only when ions are accelerated to the target material surface with hundreds of volts of energy, whereas the floating potential of nanoparticles in LTPs is only a few volts.^51,52^ Thermal evaporation of Mg particles within the plasma is a more likely explanation for the presence of atomically charged Mg in the reactor. Condensed particles can experience intense heating through surface reactions in LTPs, which we have found can heat the Mg particles to their evaporation temperature.^53−55^

**Figure 8:**
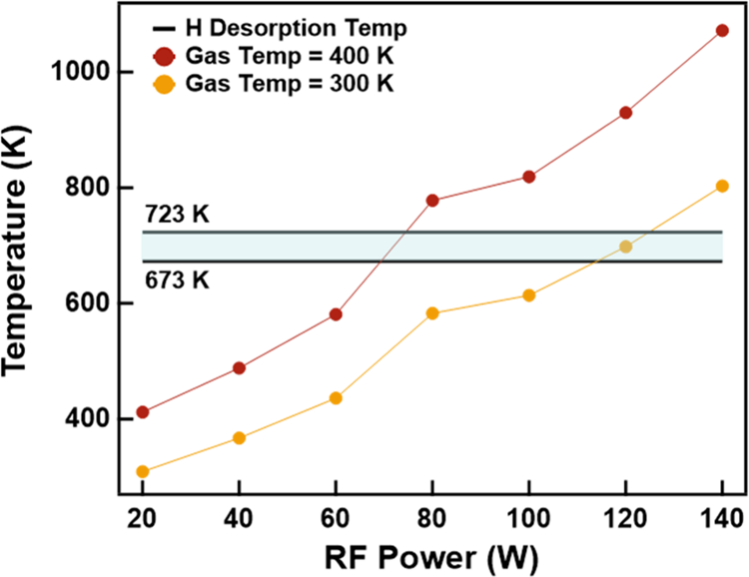
Graph of nanoparticle temperature based on the particle energy balance approach when assuming an electron temperature of 5 eV and two gas temperatures of 300 and 400 K. The area in blue is the range of H_2_ desorption temperatures as obtained by TPD (see Figure S1).

Further insights are obtained by solving a particle energy balance (eq 3) and estimating the particle temperatures at various RF powers, using the approach described by Mangolini et al.^56^ In this approach, a steady-state particle temperature is calculated by balancing the heat generation terms, induced by reactions between the particle and plasma-produced species and the cooling to the background gas. The nanoparticle heating (*G*) and cooling (*L*) terms are calculated by eqs 4 and 5, respectively. Electron–ion recombination and hydrogen recombination at the particle surface account for particle heating, whereas thermal conduction to the background gas causes particle cooling. Electron–ion recombination events at the nanoparticle surface generate heat with an energy release equal to the ionization energy of Ar (15.76 eV) for each impinging ion.^30^ The ion density was measured for a similar system to be 10^11^ to 10^12^ cm^–3^ according to the input RF power.^42^ For our calculations of electron–ion recombination heat generation, we assumed the ion density to be 10^11^ cm^–3^. Chemical reactions involving hydrogen at the particle surface also release heat and increase the particle temperature. Among them, atomic hydrogen recombination at the surface releases the most heat with an energy of 4.5 eV per atomic hydrogen pair.^54^Figure 8 shows the particle temperature computed from the energy balance using the measured H densities (Figure 7b) at various RF powers. While the gas temperature in LTPs remains low, some gas heating does occur. We, therefore, compute the nanoparticle temperature for background gas temperatures of 300 and 400 K. An electron temperature of 5 eV was used for the calculations. The model suggests that particle temperatures can surpass 1000 K at a high RF power, exceeding not only the Mg evaporation temperature but the H desorption temperature as well (Figure S1).^57−59^ This indicates that there may be excessive particle heating within the plasma at an RF power above 60 W, hindering MgH_2_ production due to H_2_ desorption from the particles. Excessive particle heating is a likely explanation for the plateauing in the MgH_2_ fraction observed from XRD when increasing the RF power (Figure 2b).
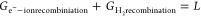
3
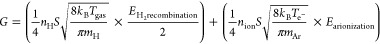
4
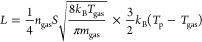
5

## 4 Conclusions

Low-temperature plasmas can process Mg NPs in-flight with hydrogen to give nanomaterials with a significant fraction of MgH_2_. Careful TEM characterization shows that larger particles (∼300 nm) have a hydrogen-rich “crust” with MgH_2_ domains in the outer shells of the particles. Smaller particles (∼50 nm) appear to be hydrogenated throughout the volume. This is consistent with an inward hydrogen diffusion process where the plasma provides an effective source of atomic hydrogen that can readily dissolve into the Mg lattice. The in-flight plasma treatment has profound effects on the combustion kinetics. The ignition temperature with the KClO_4_ oxidizer is lowered significantly upon hydrogenation, from ∼690 to ∼480 °C. The lower ignition temperature correlates well with the desorption of H_2_ from MgH_2_ in the 400–500 °C range, suggesting that the released hydrogen is responsible for effectively jumpstarting the combustion. The earlier onset of the exothermic reaction, in turn, results in the earlier release of Mg vapor, as observed by T-jump/TOFMS measurements, further enhancing the combustion of the Mg fuel. We have also performed careful characterization of the plasma process by optical emission spectroscopy. This confirms that the plasma is effective at dissociating molecular hydrogen and driving the in-flight hydrogenation of the Mg particles. On the other hand, the nanoparticle energy balance shows that the particle temperature can be sufficiently high not only to desorb hydrogen but also to initiate the evaporation of Mg. This is consistent with the fraction of MgH_2_ reaching a plateau and with the loss of Mg to the reactor walls at high plasma input powers. These insights suggest that excessive plasma power is detrimental to the treatment of Mg particles, providing a direction toward further process optimization. Overall, this work demonstrates that a rapid, in-flight, nonthermal plasma processing step can have significant effects on the combustion of Mg-based energetic nanoparticles, thus providing an additional lever to tune their energy release profile.

## Supporting Information Available

TPD measurement to experimentally measure the mmol of H_2_ desorbed from the h-Mg NPs; production rate at varying RF power and images of the film produced on a plasma reactor tube at a high RF power; size distribution of the h-Mg NPs produced using 90 and 80 A current; OES measurements showing the Ar, Mg, and H emission lines according to the RF power; information on the calculations for estimating the atomic H densities; and equations showing the calculations for mmol of H_2_ (PDF)

## References

[ref1] Calizzi M.; Venturi F.; Ponthieu M.; Cuevas F.; Morandi V.; Perkisas T.; Bals S.; Pasquini L. Gas-Phase Synthesis of Mg–Ti Nanoparticles for Solid-State Hydrogen Storage. Phys. Chem. Chem. Phys. 2016, 18 (1), 141–148. 10.1039/C5CP03092G. 26603765

[ref2] Zhang X. L.; Liu Y. F.; Zhang X.; Hu J. J.; Gao M. X.; Pan H. G. Empowering Hydrogen Storage Performance of MgH2 by Nanoengineering and Nanocatalysis. Materials Today Nano 2020, 9, 100064 10.1016/j.mtnano.2019.100064.

[ref3] Sadhasivam T.; Kim H.-T.; Jung S.; Roh S.-H.; Park J.-H.; Jung H.-Y. Dimensional Effects of Nanostructured Mg/MgH2 for Hydrogen Storage Applications: A Review. Renewable Sustainable Energy Rev. 2017, 72, 523–534. 10.1016/j.rser.2017.01.107.

[ref4] Ismail M. Effect of LaCl3 Addition on the Hydrogen Storage Properties of MgH2. Energy 2015, 79, 177–182. 10.1016/j.energy.2014.11.001.

[ref5] Maggi F.; Gariani G.; Galfetti L.; DeLuca L. T. Theoretical Analysis of Hydrides in Solid and Hybrid Rocket Propulsion. Int. J. Hydrogen Energy 2012, 37 (2), 1760–1769. 10.1016/j.ijhydene.2011.10.018.

[ref6] Wu X.-l.; Xu S.; Pang A.-m.; Cao W.-g.; Liu D.-b.; Zhu X.-y.; Xu F.-y.; Wang X. Hazard Evaluation of Ignition Sensitivity and Explosion Severity for Three Typical MH2 (M= Mg, Ti, Zr) of Energetic Materials. Def. Technol. 2021, 17 (4), 1262–1268. 10.1016/j.dt.2020.06.011.

[ref7] Reddy S. N.; Nanda S.; Vo D.-V. N.; Nguyen T. D.; Nguyen V.-H.; Abdullah B.; Nguyen-Tri P. 1-Hydrogen: Fuel of the Near Future. In New Dimensions in Production and Utilization of Hydrogen; Nanda S.; Vo D.-V. N.; Nguyen-Tri P., Eds.; Elsevier, 2020; pp 1–20.

[ref8] Xi J.; Liu J.; Wang Y.; Liang D.; Zhou J. Effect of Metal Hydrides on the Burning Characteristics of Boron. Thermochim. Acta 2014, 597, 58–64. 10.1016/j.tca.2014.10.017.

[ref9] Fang H.; Deng P.; Liu R.; Han K.; Zhu P.; Nie J.; Guo X. Energy-Releasing Properties of Metal Hydrides (MgH2, TiH2 and ZrH2) with Molecular Perovskite Energetic Material DAP-4 as a Novel Oxidant. Combust. Flame 2023, 247, 112482 10.1016/j.combustflame.2022.112482.

[ref10] Young G.; Piekiel N.; Chowdhury S.; Zachariah M. R. Ignition Behavior of α-AlH3. Combust. Sci. Technol. 2010, 182 (9), 1341–1359. 10.1080/00102201003694834.

[ref11] Zaluska A.; Zaluski L.; Ström–Olsen J. O. Nanocrystalline Magnesium for Hydrogen Storage. J. Alloys Compd. 1999, 288 (1), 217–225. 10.1016/S0925-8388(99)00073-0.

[ref12] Wu J.-x.; Liu Q.; Feng B.; Yin Q.; Li Y.-c.; Wu S.-z.; Yu Z.-s.; Huang J.-y.; Ren X.-x. Improving the Energy Release Characteristics of PTFE/Al by Doping Magnesium Hydride. Def. Technol. 2022, 18 (2), 219–228. 10.1016/j.dt.2020.12.008.

[ref13] Yetter R. A. Progress Towards Nanoengineered Energetic Materials. Proc. Combust. Inst. 2021, 38 (1), 57–81. 10.1016/j.proci.2020.09.008.

[ref14] Perez J. P. L.; McMahon B. W.; Yu J.; Schneider S.; Boatz J. A.; Hawkins T. W.; McCrary P. D.; Flores L. A.; Rogers R. D.; Anderson S. L. Boron Nanoparticles with High Hydrogen Loading: Mechanism for B–H Binding and Potential for Improved Combustibility and Specific Impulse. ACS Appl. Mater. Interfaces 2014, 6 (11), 8513–8525. 10.1021/am501384m. 24806745

[ref15] Ghildiyal P.; Ke X.; Biswas P.; Nava G.; Schwan J.; Xu F.; Kline D. J.; Wang H.; Mangolini L.; Zachariah M. R. Silicon Nanoparticles for the Reactivity and Energetic Density Enhancement of Energetic-Biocidal Mesoparticle Composites. ACS Appl. Mater. Interfaces 2021, 13 (1), 458–467. 10.1021/acsami.0c17159. 33373186

[ref16] Jiang Y.; Wang Y.; Baek J.; Wang H.; Gottfried J. L.; Wu C.-C.; Shi X.; Zachariah M. R.; Zheng X. Ignition and Combustion of Perfluoroalkyl-functionalized Aluminum Nanoparticles and Nanothermite. Combust. Flame 2022, 242, 112170 10.1016/j.combustflame.2022.112170.

[ref17] Young G.; Wang H.; Zachariah M. R. Application of Nano-Aluminum/Nitrocellulose Mesoparticles in Composite Solid Rocket Propellants. Propellants, Explos., Pyrotech. 2015, 40 (3), 413–418. 10.1002/prep.201500020.

[ref18] Mulamba O.; Hunt E. M.; Pantoya M. L. Neutralizing Bacterial Spores Using Halogenated Energetic Reactions. Biotechnol. Bioprocess Eng. 2013, 18 (5), 918–925. 10.1007/s12257-013-0323-3.

[ref19] Zhang Q.; Huang Y.; Ma T.; Li K.; Ye F.; Wang X.; Jiao L.; Yuan H.; Wang Y. Facile Synthesis of Small MgH2 Nanoparticles Confined in Different Carbon Materials for Hydrogen Storage. J. Alloys Compd. 2020, 825, 153953 10.1016/j.jallcom.2020.153953.

[ref20] Paskevicius M.; Sheppard D. A.; Buckley C. E. Thermodynamic Changes in Mechanochemically Synthesized Magnesium Hydride Nanoparticles. J. Am. Chem. Soc. 2010, 132 (14), 5077–5083. 10.1021/ja908398u. 20307102

[ref21] Porcheddu A.; Cincotti A.; Delogu F. Kinetics of MgH2 Formation by Ball Milling. Int. J. Hydrogen Energy 2021, 46 (1), 967–973. 10.1016/j.ijhydene.2020.09.251.

[ref22] Ouyang L.; Cao Z.; Wang H.; Hu R.; Zhu M. Application of Dielectric Barrier Discharge Plasma-Assisted Milling in Energy Storage Materials – A Review. J. Alloys Compd. 2017, 691, 422–435. 10.1016/j.jallcom.2016.08.179.

[ref23] Li Z.-Y.; Sun Y.-J.; Zhang C.-C.; Wei S.; Zhao L.; Zeng J.-L.; Cao Z.; Zou Y.-J.; Chu H.-L.; Xu F.; Sun L.-X.; Pan H.-G. Optimizing Hydrogen Ad/Desorption of Mg-Based Hydrides for Energy-Storage Applications. J. Mater. Sci. Technol. 2023, 141, 221–235. 10.1016/j.jmst.2022.08.047.

[ref24] Klopčič N.; Grimmer I.; Winkler F.; Sartory M.; Trattner A. A Review on Metal Hydride Materials for Hydrogen Storage. J. Energy Storage 2023, 72, 108456 10.1016/j.est.2023.108456.

[ref25] Song M.; Zhang L.; Wu F.; Zhang H.; Zhao H.; Chen L.; Li H. Recent Advances of Magnesium Hydride as an Energy Storage Material. J. Mater. Sci. Technol. 2023, 149, 99–111. 10.1016/j.jmst.2022.11.032.

[ref26] Ouyang L. Z.; Cao Z. J.; Wang H.; Liu J. W.; Sun D. L.; Zhang Q. A.; Zhu M. Enhanced Dehydriding Thermodynamics and Kinetics in Mg(In)–MgF2 Composite Directly Synthesized by Plasma Milling. J. Alloys Compd. 2014, 586, 113–117. 10.1016/j.jallcom.2013.10.029.

[ref27] Dan L.; Wang H.; Liu J.; Ouyang L.; Zhu M. H2 Plasma Reducing Ni Nanoparticles for Superior Catalysis on Hydrogen Sorption of MgH2. ACS Appl. Energy Mater. 2022, 5 (4), 4976–4984. 10.1021/acsaem.2c00206.

[ref28] Le-Quoc H.; Lacoste A.; Miraglia S.; Béchu S.; Bès A.; Laversenne L. MgH2 Thin Films Deposited by One-Step Reactive Plasma Sputtering. Int. J. Hydrogen Energy 2014, 39 (31), 17718–17725. 10.1016/j.ijhydene.2014.08.096.

[ref29] Le-Quoc H.; Coste M.; Lacoste A.; Laversenne L. Magnesium Hydride Films Deposited on Flexible Substrates: Structure, Morphology and Hydrogen Sorption Properties. J. Alloys Compd. 2023, 955, 170272 10.1016/j.jallcom.2023.170272.

[ref30] Mangolini L.; Thimsen E.; Kortshagen U. High-Yield Plasma Synthesis of Luminescent Silicon Nanocrystals. Nano Lett. 2005, 5 (4), 655–659. 10.1021/nl050066y. 15826104

[ref31] Izadi A.; Anthony R. J. A Plasma-Based Gas-Phase Method for Synthesis of Gold Nanoparticles. Plasma Processes Polym. 2019, 16 (7), e1800212 10.1002/ppap.201800212.

[ref32] Loh K. Q.; Andaraarachchi H. P.; Ferry V. E.; Kortshagen U. R. Photoluminescent Si/SiO2 Core/Shell Quantum Dots Prepared by High-Pressure Water Vapor Annealing for Solar Concentrators, Light-Emitting Devices, and Bioimaging. ACS Appl. Nano Mater. 2023, 6 (7), 6444–6453. 10.1021/acsanm.3c01130.

[ref33] Beaudette C. A.; Andaraarachchi H. P.; Wu C.-C.; Kortshagen U. R. Inductively Coupled Nonthermal Plasma Synthesis of Aluminum Nanoparticles. Nanotechnology 2021, 32 (39), 395601 10.1088/1361-6528/ac0cb3. 34144546

[ref34] Barragan A. A.; Hanukovich S.; Bozhilov K.; Yamijala S. S. R. K. C.; Wong B. M.; Christopher P.; Mangolini L. Photochemistry of Plasmonic Titanium Nitride Nanocrystals. J. Phys. Chem. C 2019, 123 (35), 21796–21804. 10.1021/acs.jpcc.9b06257.

[ref35] Uner N. B.; Thimsen E. Nonequilibrium Plasma Aerotaxy of Size Controlled GaN Nanocrystals. J. Phys. D: Appl. Phys. 2020, 53 (9), 095201 10.1088/1361-6463/ab59e6.

[ref36] Kortshagen U.; Bhandarkar U. Modeling of Particulate Coagulation in Low Pressure Plasmas. Phys. Rev. E 1999, 60 (1), 887 10.1103/PhysRevE.60.887. 11969833

[ref37] Graves D. B.; Daugherty J. E.; Kilgore M. D.; Porteous R. K. Charging, Transport and Heating of Particles in Radiofrequency and Electron Cyclotron Resonance Plasmas. Plasma Sources Sci. Technol. 1994, 3 (3), 433 10.1088/0963-0252/3/3/029.

[ref38] Wagner B.; Ghildiyal P.; Biswas P.; Chowdhury M.; Zachariah M. R.; Mangolini L. In-Flight Synthesis of Core–Shell Mg/Si–SiOx Particles with Greatly Reduced Ignition Temperature. Adv. Funct. Mater. 2023, 33, 2212805 10.1002/adfm.202212805.

[ref39] Xu F.; Nava G.; Biswas P.; Dulalia I.; Wang H.; Alibay Z.; Gale M.; Kline D. J.; Wagner B.; Mangolini L.; Zachariah M. R. Energetic Characteristics of Hydrogenated Amorphous Silicon Nanoparticles. Chem. Eng. J. 2022, 430, 133140 10.1016/j.cej.2021.133140.

[ref40] Agarwal P. P. K.; Matsoukas T. Engineered Surface Chemistry and Enhanced Energetic Performance of Aluminum Nanoparticles by Nonthermal Hydrogen Plasma Treatment. Nano Lett. 2023, 23 (12), 5541–5547. 10.1021/acs.nanolett.3c00908. 37289964

[ref41] Ringe E. Shapes, Plasmonic Properties, and Reactivity of Magnesium Nanoparticles. J. Phys. Chem. C 2020, 124 (29), 15665–15679. 10.1021/acs.jpcc.0c03871. PMC7467285 32905178

[ref42] Uner N. B.; Thimsen E. In-Flight Size Focusing of Aerosols by a Low Temperature Plasma. J. Phys. Chem. C 2017, 121 (23), 12936–12944. 10.1021/acs.jpcc.7b03572.

[ref43] Ghildiyal P.; Biswas P.; Herrera S.; Xu F.; Alibay Z.; Wang Y.; Wang H.; Abbaschian R.; Zachariah M. R. Vaporization-Controlled Energy Release Mechanisms Underlying the Exceptional Reactivity of Magnesium Nanoparticles. ACS Appl. Mater. Interfaces 2022, 14 (15), 17164–17174. 10.1021/acsami.1c22685. 35390252

[ref44] Borgschulte A.; Bösenberg U.; Barkhordarian G.; Dornheim M.; Bormann R. Enhanced Hydrogen Sorption Kinetics of Magnesium by Destabilized MgH2−δ. Catal. Today 2007, 120 (3), 262–269. 10.1016/j.cattod.2006.09.031.

[ref45] NIST Atomic Spectra Database (version 5.10). https://www.nist.gov/pml/atomic-spectra-database. (accessed 2023–01–20).

[ref46] Hagelaar G. J. M.; Pitchford L. C. Solving the Boltzmann Equation to Obtain Electron Transport Coefficients and Rate Coefficients for Fluid Models. Plasma Sources Sci. Technol. 2005, 14 (4), 722 10.1088/0963-0252/14/4/011.

[ref47] Mosbach T. Population Dynamics of Molecular Hydrogen and Formation of Negative Hydrogen Ions in a Magnetically Confined Low Temperature Plasma. Plasma Sources Sci. Technol. 2005, 14 (3), 610 10.1088/0963-0252/14/3/026.

[ref48] Sabat K. C.; Murphy A. B. Hydrogen Plasma Pocessing of Iron Ore. Metall. Mater. Trans. B 2017, 48 (3), 1561–1594. 10.1007/s11663-017-0957-1.

[ref49] Celiberto R.; Janev R.; Laricchiuta A.; Capitelli M.; Wadehra J.; Atems D. Cross Section Data for Electron-Impact Inelastic Processes of Vibrationally Excited Molecules of Hydrogen and its Isotopes. At. Data Nucl. Data Tables 2001, 77 (2), 161–213. 10.1006/adnd.2000.0850.

[ref50] Tao S. X.; Notten P. H. L.; van Santen R. A.; Jansen A. P. J. Density Functional Theory Studies of the Hydrogenation Properties of Mg and Ti. Phys. Rev. B 2009, 79 (14), 144121 10.1103/PhysRevB.79.144121.

[ref51] Thornton J. A. Magnetron Sputtering: Basic Physics and Application to Cylindrical Magnetrons. J. Vac. Sci. Technol. 1978, 15 (2), 171–177. 10.1116/1.569448.

[ref52] Gatti M.; Kortshagen U. Analytical Model of Particle Charging in Plasmas over a Wide Range of Collisionality. Phys. Rev. E 2008, 78 (4), 046402 10.1103/PhysRevE.78.046402. 18999538

[ref53] Coleman D.; Lopez T.; Yasar-Inceoglu O.; Mangolini L. Hollow Silicon Carbide Nanoparticles from a Non-Thermal Plasma Process. J. Appl. Phys. 2015, 117 (19), 193301 10.1063/1.4919918.

[ref54] Kramer N. J.; Anthony R.; Mamunuru M.; Aydil E.; Kortshagen U. Plasma-Induced Crystallization of Silicon Nanoparticles. J. Phys. D: Appl. Phys. 2014, 47 (7), 075202 10.1088/0022-3727/47/7/075202.

[ref55] Gulbransen E. A. The Oxidation and Evaporation of Magnesium at Temperatures from 400 to 500 C. Trans. Electrochem. Soc. 1945, 87 (1), 589 10.1149/1.3071667.

[ref56] Mangolini L.; Kortshagen U. Selective Nanoparticle Heating: Another Form of Nonequilibrium in Dusty Plasmas. Phys. Rev. E 2009, 79 (2), 026405 10.1103/PhysRevE.79.026405. 19391853

[ref57] Shao H.; Wang Y.; Xu H.; Li X. Hydrogen Storage Properties of Magnesium Ultrafine Particles Prepared by Hydrogen Plasma-Metal Reaction. Mater. Sci. Eng.: B 2004, 110 (2), 221–226. 10.1016/j.mseb.2004.03.013.

[ref58] Xie L.; Liu Y.; Wang Y. T.; Zheng J.; Li X. G. Superior Hydrogen Storage Kinetics of MgH2 Nanoparticles Doped with TiF3. Acta Mater. 2007, 55 (13), 4585–4591. 10.1016/j.actamat.2007.04.020.

[ref59] Bösenberg U.; Ravnsbæk D. B.; Hagemann H.; D’Anna V.; Minella C. B.; Pistidda C.; van Beek W.; Jensen T. R.; Bormann R.; Dornheim M. Pressure and Temperature Influence on the Desorption Pathway of the LiBH4–MgH2 Composite System. J. Phys. Chem. C 2010, 114 (35), 15212–15217. 10.1021/jp104814u.

