# Supplementary material for: Enhancing the Combustion of Magnesium Nanoparticles via Low-Temperature Plasma-Induced Hydrogenation
Source: ACS Appl Mater Interfaces. 2023 Oct 30;15(44):51639–49. doi: 10.1021/acsami.3c12696 (PMC10636712; doi:10.1021/acsami.3c12696)
Supplement: Supplementary file 1 — am3c12696_si_001.pdf [file am3c12696_si_001.pdf]

<u>Supporting Information for:</u>

Enhancing the Combustion of Magnesium Nanoparticles via Low-Temperature Plasma-Induced Hydrogenation

†*Brandon Wagner*[1], †*Minseok Kim*[2], *Mahbub Chowdhury*[3], *Emmanuel Vidales Pasos*[2], *Kimberly Hizon*[2], *Pankaj Ghildiyal*[3], *Michael R. Zachariah*[3*], *Lorenzo Mangolini*[1,2*]

† Equal Contribution: These authors contributed equally to this work.

* mrz@engr.ucr.edu; lmangolini@engr.ucr.edu

1 Materials Science and Engineering Program, University of California Riverside, 900 University Avenue, Riverside, California 92521, United States.

2 Department of Mechanical Engineering, University of California Riverside, 900 University Avenue, Riverside, California 92521, United States.

3 Department of Chemical and Environmental Engineering, University of California Riverside,

900 University Avenue, Riverside, California 92521, United States.

Keywords: Magnesium, non-thermal plasma, hydrogen treatment, magnesium hydride, combustion, ignition, energetics

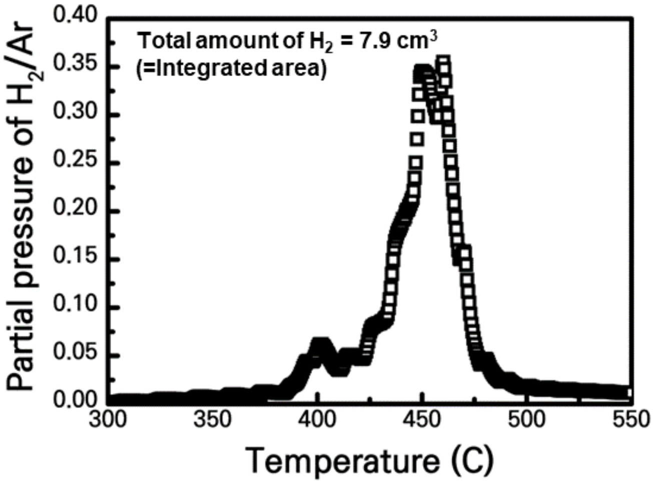

Figure s1: TPD of 55 mg h-Mg NPs with a heating rate of 10 ^{o}C min^{-1}. The integrated area underneath the curve yielded the volume of H_2 gas.

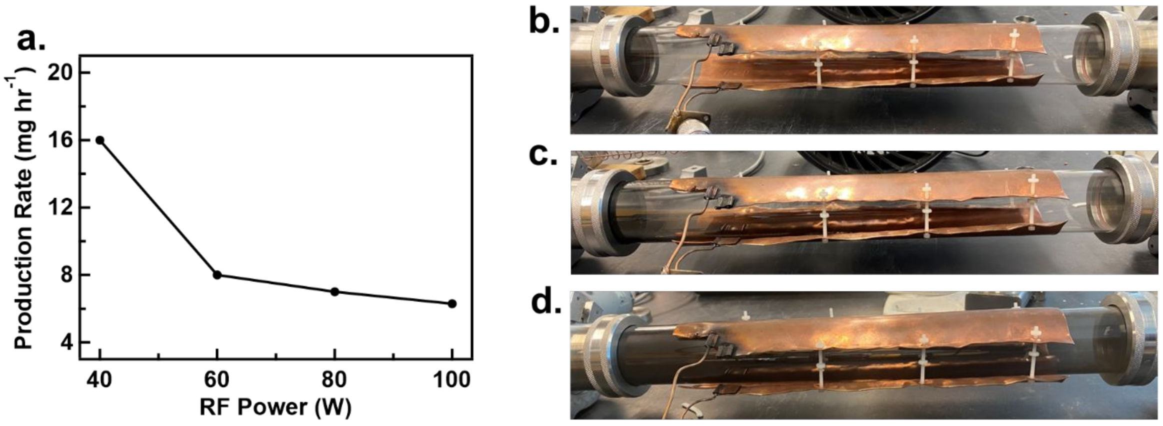

Figure s2: (a) Production rates of h-Mg NPs with RF power varied from 40 W to 100 W. (b) A photograph of the plasma reactor before synthesis. Photographs of the plasma reactor after synthesis at (c) 80 W and (d) 100 W of RF power.

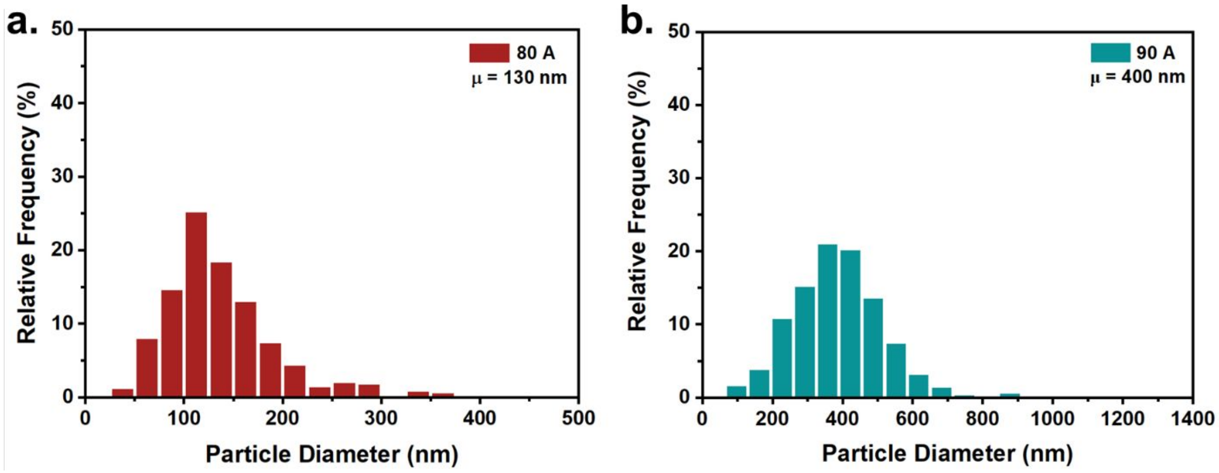

Figure s3: Particle size distributions of h-Mg NPs synthesized using (a) 80 A and (b) 90 A of current.

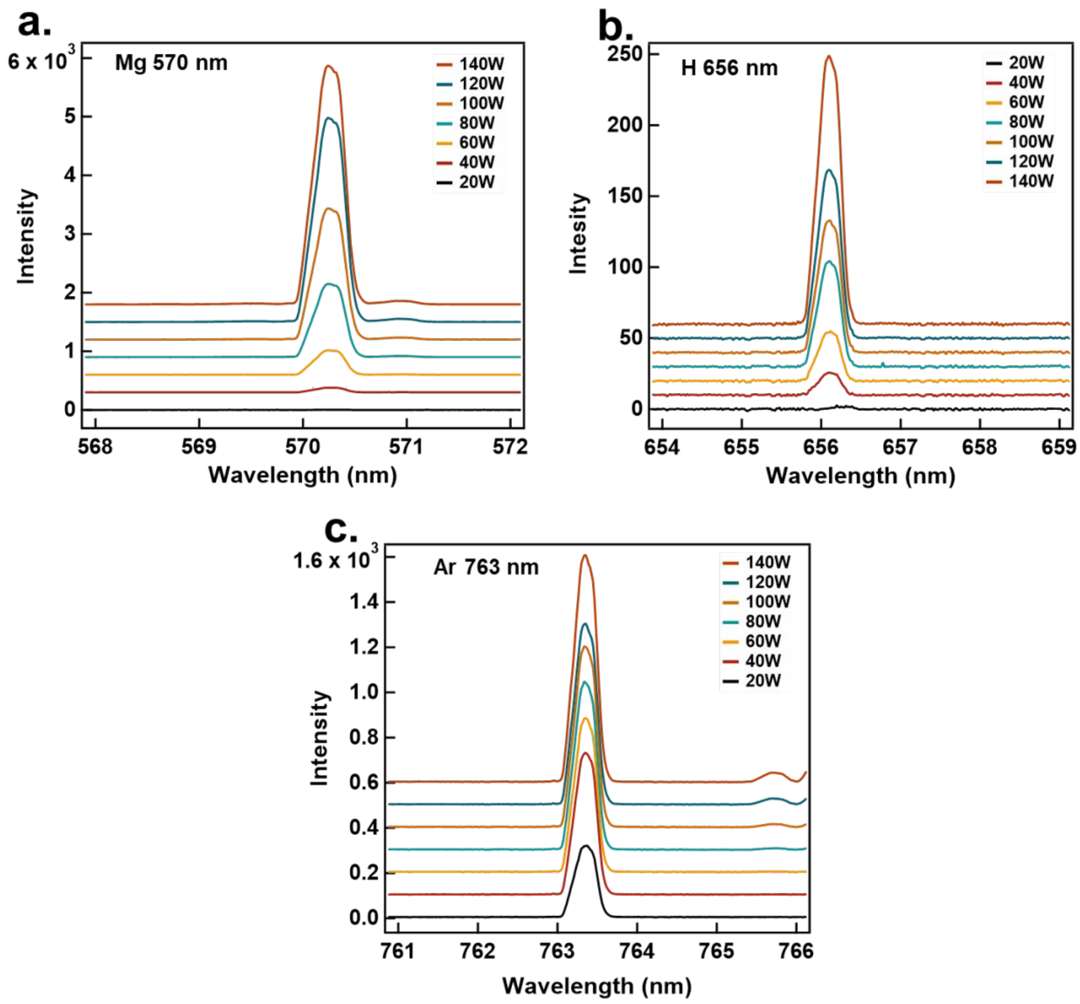

Figure s4: OES measurements showing the emission lines of (a) Mg 570 nm, (b) H 656 nm, and (c) Ar 763 nm.

Actinometry to Estimate Hydrogen Density:

An actinometry was employed to estimate the atomic hydrogen density in the plasma. By utilizing the intensity ratio of emission lines obtained from our optical emission spectroscopy (OES) measurements, a relationship summarized by Tatarova et al. is used to derive the atomic hydrogen density[1]:

$$\frac{n_H}{n_{Ar}} = \frac{I(656)}{I(763)} \times \frac{656}{763}\left(\frac{X_{Ar}}{X_H}\right) \times \frac{A_{750}/(\sum A_{j,Ar} + K_M^{Ar} \times n_M)}{A_{656}/\sum A_{j,H}}$$

where n_H and n_{Ar} are the densities of atomic hydrogen and argon, respectively. n_{Ar} was obtained by the ideal gas law. The emission intensities $I(656)$ and $I(763)$ represent the respective atomic species of hydrogen and argon at those specific wavelengths. The following emission lines were used to derive atomic hydrogen density: Ar line at 763.5 nm (transition 4p to 4s) and H line at 656.2 nm (transition from $3d^2D$ to $2P^2P_0$). A_{656} and A_{750} are the transition probabilities for the corresponding lines. $A_{j,Ar}$ and $A_{j,H}$ are the transition probabilities for all other optically allowed transitions from the same excited states for the corresponding atomic species. It is necessary to achieve the accurate branching ratio. The transition probabilities used in this analysis are sourced from the NIST atomic spectra database.[2] K_M^{Ar} is quenching rate that takes into consideration the nonradiative relaxation of the excited state for Ar. The variable n_M is the density of the collision partner, which is determined using the ideal gas law. (M=Ar and H_2) For the specific numerical values in this relation, we refer the reader to our previous report.[3] X_{Ar} and X_H represent the rates of excitation by electron impact to the selected states of Ar and H. These rates were obtained through BOLSIG+ calculation, a commonly used software for the solution of the Boltzmann transport equation.[4] The collision cross-section for Ar, H, and H_2 are acquired from the LXCat database.[5-7] Despite the dependence of excitation rates on the electron temperature, the derived atomic hydrogen density as function of plasma power shows almost same trend according to the electron temperature since emission line intensities are strongly influenced by the plasma power. Uner et al. actually measured electron temperature through quite similar plasma reactor with our reactor, and it was 4±1.5 eV.[8] Therefore, based on our previous estimation for electron temperature and reactor geometry that used in this study, we assumed that the electron temperature is 5 eV.[9]

Details of the calculation of the hydrogen content

For the TPD measurement, we used 55 mg of h-Mg sample with 17.8 wt.% content according to Rietveld Refinement fitting method. **Figure s5** shows the fitting of the XRD pattern. Profex software uses an algorithm to calculate cell parameters and peak positions of a space group. The peak intensities are then obtained from calculated structure factors from the atomic sites of a certain phase. The peak position on the 2theta axis is matched by optimizing the cell parameters while the intensities are matched by changing the scale factor. After the fitting, the weight fraction of a phase is calculated by the following equation:

$$W_a = \frac{S_a * (Z * M * V)_a}{\sum S_i * (Z * M * V)_i}$$

W is the weight percent of the phase, S is the Rietveld scale factor, Z is the number formula units per unit cell, M is the mass of the formula unit, and V is the volume of the unit cell.[10] We used the program's fitting method through their algorithm to help quantify the relative phase composition of MgH_2 from the h-Mg sample.

The TPD measurements provided us the flow rate of H_2 according to time upon heating the 55 mg of h-Mg NPs in a furnace (**Figure s1**). This can be simply done because the argon flow rate through the furnace is well known. By integrating the area underneath the peak, we obtained the total volume of H_2 desorbed from the particles. The following equations helped estimate the mmol of H_2 from the weight percentage obtained by Rietveld Refinement and from the experimental measurement from TPD. We added this information to the supporting information document.

<u>Expected mmol of H_2 according to Rietveld Refinement phase composition:</u>

$$\frac{17.8\, wt.\%\ of\ MgH_2 \div 26\frac{g}{mol}}{\left(17.8\, wt.\%\ of\ MgH_2 \div 26\frac{g}{mol}\right) + (82.2\, wt.\%\ of\ Mg \div 24\frac{g}{mol})} = 16.7\ at.\%$$

$$55\ mg * 17.8\ wt.\%\ MgH_2 = 9.79\ mg\ of\ MgH_2$$

$$\frac{9.79\ mg * 7.6\ wt.\%\ H_2\ in\ MgH_2}{2\frac{g}{mol}} = 0.372\ mmol\ of\ H_2$$

<u>Experimental mmol of H_2 observed by TPD:</u>

$$\frac{7.9\ cm^3\ of\ H_2 * 0.08988\frac{g}{L}\ H_2\ density}{2\frac{g}{mol}} = 0.355\ mmol\ of\ H_2$$

Reference

[1] Tatarova, E.; Dias, F. M.; Gordiets, B.; Ferreira, C. M. Molecular dissociation in N_2-H_2 microwave discharges. Plasma Sources Sci. Technol. 2005, 14, 19−31.

[2] NIST Standard Reference Database 78, ver 5.10; https://www. nist.gov/pml/atomic-spectra-database (accessed in 2023-01-20).

[3] Yamijala, S. S. R. K. C.; Nava, G.; Ali, Z. A.; Beretta, D.; Wong, B. M.; Mangolini, L. Harnessing Plasma Environments for Ammonia Catalysis: Mechanistic insights from experiments and large-scale Ab Initio molecular dynamics. J. Phys. Chem. Lett. 2020, 11, 10469− 10475.

[4] Hagelaar, G. J. M.; Pitchford, L. C. Solving the Boltzmann equation to obtain electron transport coefficients and rate coefficients for fluid models. Plasma Sources Sci. Technol. 2005, 14, 722−733.

[5] BSR database, www.lxcat.net/BSR, retrieved in 2023-01-20.

[6] IST-Lisbon database, www.lxcat.net/IST-Lisbon, retrieved in 2023-01-20.

[7] Itikawa database, www.lxcat.net/Itikawa, retrieved on 2023-01-20.

[8] Uner, N. B.; Thimsen, E. In-Flight Size Focusing of Aerosols by a Low Temperature Plasma. J. Phys. Chem. C 2017, 121, 12936− 12944.

[9] Kim, M.; Biswas, S.; Nava, G.; Wong, B. M.; Mangolini, L. Reduced energy cost of ammonia synthesis via RF plasma pulsing. ACS Sustain. Chem. Eng. 2022, 10, 15135–15147.

[10] Döbelin, N., Kleeberg, R., Profex: a graphical user interface for the Rietveld refinement program *BGMN*, Journal of Applied Crystallography 48 (2015), 1573-1580.
